# Blood DNA Methylation Patterns in Older Adults With Evolving Dementia

**DOI:** 10.1093/gerona/glac068

**Published:** 2022-03-17

**Authors:** Raúl Fernández Pérez, Juan José Alba-Linares, Juan Ramón Tejedor, Agustín Fernández Fernández, Miguel Calero, Aurora Román-Domínguez, Consuelo Borrás, José Viña, Jesús Ávila, Miguel Medina, Mario Fernández Fraga

**Affiliations:** Cancer Epigenetics and Nanomedicine Laboratory, Nanomaterials and Nanotechnology Research Center (CINN-CSIC), El Entrego, Spain; Health Research Institute of Asturias (ISPA-FINBA), University of Oviedo, Oviedo, Spain; Institute of Oncology of Asturias (IUOPA) and Department of Organisms and Systems Biology (B.O.S.), University of Oviedo, Oviedo, Spain; Rare Diseases CIBER (CIBERER) of the Carlos III Health Institute (ISCIII), Madrid, Spain; Cancer Epigenetics and Nanomedicine Laboratory, Nanomaterials and Nanotechnology Research Center (CINN-CSIC), El Entrego, Spain; Health Research Institute of Asturias (ISPA-FINBA), University of Oviedo, Oviedo, Spain; Institute of Oncology of Asturias (IUOPA) and Department of Organisms and Systems Biology (B.O.S.), University of Oviedo, Oviedo, Spain; Rare Diseases CIBER (CIBERER) of the Carlos III Health Institute (ISCIII), Madrid, Spain; Cancer Epigenetics and Nanomedicine Laboratory, Nanomaterials and Nanotechnology Research Center (CINN-CSIC), El Entrego, Spain; Health Research Institute of Asturias (ISPA-FINBA), University of Oviedo, Oviedo, Spain; Institute of Oncology of Asturias (IUOPA) and Department of Organisms and Systems Biology (B.O.S.), University of Oviedo, Oviedo, Spain; Rare Diseases CIBER (CIBERER) of the Carlos III Health Institute (ISCIII), Madrid, Spain; Cancer Epigenetics and Nanomedicine Laboratory, Nanomaterials and Nanotechnology Research Center (CINN-CSIC), El Entrego, Spain; Health Research Institute of Asturias (ISPA-FINBA), University of Oviedo, Oviedo, Spain; Institute of Oncology of Asturias (IUOPA) and Department of Organisms and Systems Biology (B.O.S.), University of Oviedo, Oviedo, Spain; Rare Diseases CIBER (CIBERER) of the Carlos III Health Institute (ISCIII), Madrid, Spain; Network Center for Biomedical Research in Neurodegenerative Diseases (CIBERNED), Madrid, Spain; Chronic Disease Programme (UFIEC), Instituto de Salud Carlos III, Madrid, Spain; CIEN Foundation, Queen Sofia Foundation Alzheimer Center, Madrid, Spain; Freshage Research Group, Department of Physiology, Faculty of Medicine, University of Valencia and CIBERFES-ISCIII, Fundación Investigación Hospital Clínico Universitario/INCLIVA, Valencia, Spain; Freshage Research Group, Department of Physiology, Faculty of Medicine, University of Valencia and CIBERFES-ISCIII, Fundación Investigación Hospital Clínico Universitario/INCLIVA, Valencia, Spain; Freshage Research Group, Department of Physiology, Faculty of Medicine, University of Valencia and CIBERFES-ISCIII, Fundación Investigación Hospital Clínico Universitario/INCLIVA, Valencia, Spain; Network Center for Biomedical Research in Neurodegenerative Diseases (CIBERNED), Madrid, Spain; Centro de Biología Molecular Severo Ochoa (CBMSO) CSIC-UAM, Madrid, Spain; Network Center for Biomedical Research in Neurodegenerative Diseases (CIBERNED), Madrid, Spain; CIEN Foundation, Queen Sofia Foundation Alzheimer Center, Madrid, Spain; Cancer Epigenetics and Nanomedicine Laboratory, Nanomaterials and Nanotechnology Research Center (CINN-CSIC), El Entrego, Spain; Health Research Institute of Asturias (ISPA-FINBA), University of Oviedo, Oviedo, Spain; Institute of Oncology of Asturias (IUOPA) and Department of Organisms and Systems Biology (B.O.S.), University of Oviedo, Oviedo, Spain; Rare Diseases CIBER (CIBERER) of the Carlos III Health Institute (ISCIII), Madrid, Spain

**Keywords:** Cognitive decline, Dementia, DNA methylation, Epigenetics, Epigenetic age

## Abstract

Dementia and cognitive disorders are major aging-associated pathologies. The prevalence and severity of these conditions are influenced by both genetic and environmental factors. Reflecting this, epigenetic alterations have been associated with each of these processes, especially at the level of DNA methylation, and such changes may help explain the observed interindividual variability in the development of the 2 pathologies. However, the importance of epigenetic alterations in explaining their etiology is unclear because little is known about the timing of when they appear. Here, using Illumina MethylationEPIC arrays, we have longitudinally analyzed the peripheral blood methylomes of cognitively healthy older adults (>70 year), some of whom went on to develop dementia while others stayed healthy. We have characterized 34 individuals at the prediagnosis stage and at a 4-year follow-up in the postdiagnosis stage (total *n* = 68). Our results show multiple DNA methylation alterations linked to dementia status, particularly at the level of differentially methylated regions. These loci are associated with several dementia-related genes, including *PON1*, *AP2A2*, *MAGI2*, *POT1*, *ITGAX, PACSIN1, SLC2A8,* and *EIF4E*. We also provide validation of the previously reported epigenetic alteration of *HOXB6* and *PM20D1*. Importantly, we show that most of these regions are already altered in the prediagnosis stage of individuals who go on to develop dementia. In conclusion, our observations suggest that dementia-associated epigenetic patterns that have specific biological features are already present before diagnosis, and thus may be important in the design of epigenetic biomarkers for disease detection based on peripheral tissues.

## Background

Cognitive decline and dementia are complex diseases in which both genetic and environmental factors play a relevant role (1,2). The well-known preclinical phenotypes of dementia (3) serve to demonstrate that these pathologies are defined by progressive changes whose timely detection is crucial in the management of the disease. Indeed, dementia is strongly associated with aging, although the causal relationships between the overlap in cognitive decline symptoms observed for the 2 processes remain to be clarified (4). Both aging and dementia have been associated with epigenetic alterations, and these molecular mechanisms may contribute to characterizing their relationship (5). During aging, both genetic factors and the accumulation of external stimuli, such as those related to lifestyle, can trigger epigenetic changes which may help explain: (a) the variability in the trajectories of cognitive decline experienced by “healthy” aging individuals (6) and (b) the variability in the appearance of pathological states such as mild cognitive impairment or dementia (5).

Among the known epigenetic changes, DNA methylation alterations have been found to be associated with dementia both in brain and in systemic tissues such as peripheral blood (7). In addition, the recently developed DNA methylation clocks, which are often altered in disease, can serve as proxies that encompass the complex factors (genetic, biological, and environmental) which lead to interindividual differences in phenotype and are thus of great interest in the definition of potential biomarkers of disease (8). Nonetheless, the question still remains as to whether these epigenetic alterations arise prior to or as a consequence of dementia. If the former, they could serve as biological indicators and/or provide novel avenues for interventions to prevent these diseases. Within this scenario, longitudinal studies are of great value in tracing variables that contribute to explaining these phenotypes (9).

## Method

**Figure 1.**
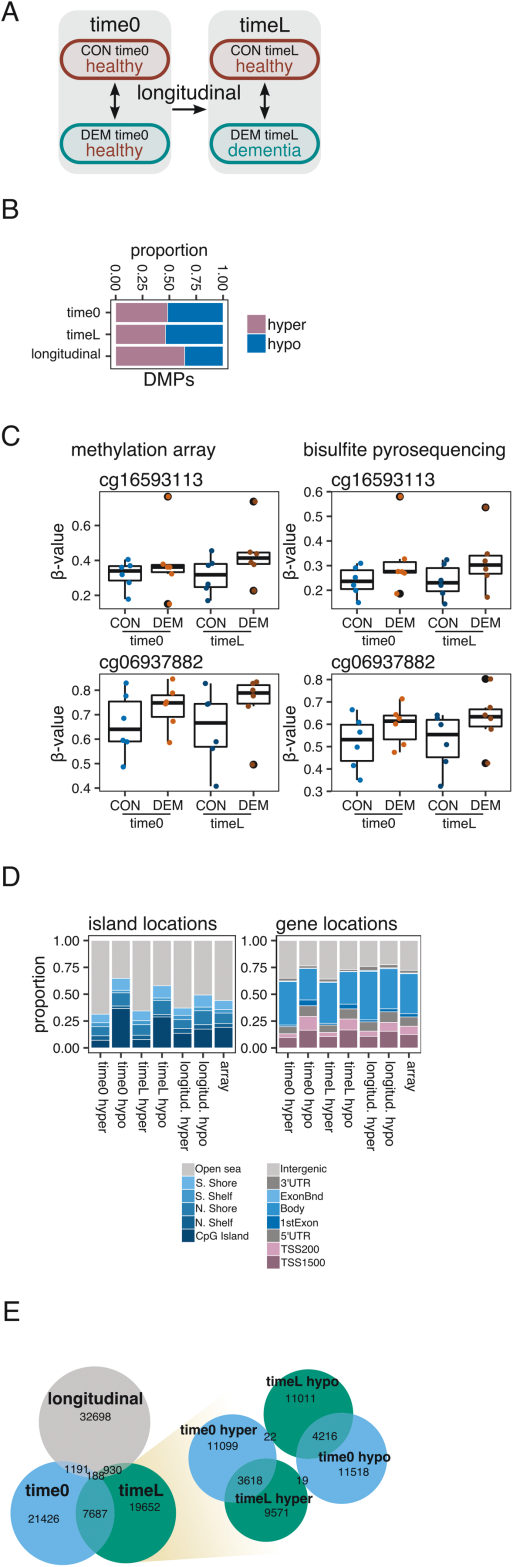
DNA methylation alterations at single-CpG sites in dementia. (A) Schematic of the study design. (B) Barplots depicting the proportion of hyper- and hypo-methylated DMPs (unadjusted *p* < .05) found in the time0, timeL, and longitudinal comparisons. (C) Boxplots comparing the DNA methylation measurements performed by the Infinium MethylationEPIC BeadChip and by bisulfite pyrosequencing for 2 CpGs (cg16593113, cg06937882) on a subset of 24 samples segregated by experimental group. (D) Barplots showing the relative distribution of hyper- and hypo-methylated DMPs in the time0, timeL, and longitudinal comparisons according to their CpG island location status (top) and gene location status (bottom). The rightmost bars reflect the background distribution of all the analyzed array probes. (E) On the left, the Venn diagram describes the numbers and intersections of the DMPs found for the time0, timeL, and longitudinal comparisons. On the right, the Venn diagram shows the specific intersections between hyper- and hypo-methylated DMPs from the time0 to timeL comparisons. CON = control; DEM = dementia; DMPs = differentially methylated probes.

Here, we have profiled the peripheral blood mononuclear cell epigenome of 68 samples at more than 770 000 CpG sites by employing Infinium MethylationEPIC BeadChips. We studied a longitudinal cohort of older adults consisting of 17 pairs of age-matched, cognitively healthy individuals where in a 4-year follow-up assessment (*SD* = 0.35 year), one was still cognitively healthy, that is acted as control (CON), while the other had been diagnosed with dementia (DEM, also referred to henceforth as “converter” individuals; Figure 1a, Table 1; Supplementary Table 1 for extended phenotypic data). Subjects are volunteer participants in an ongoing single-center longitudinal study known as “The Vallecas Project” where they annually undergo extensive neurological and neuropsychological assessment (10). We characterized the methylomes of these individuals at an initial, prediagnosis time point, when all were cognitively healthy (time0; CON_time0, and DEM_time0 groups, *n* = 34) and at a longitudinal, postdiagnosis time point at which some had converted to dementia (timeL; CON_timeL, and DEM_timeL groups, *n* = 34). This allowed us to examine: (a) DNA methylation alterations predictive of the appearance of cognitive pathology (time0 comparison), (b) DNA methylation alterations directly associated with cognitive pathology (timeL comparison), and (c) longitudinal DNA methylation alterations (the full methodology is detailed in Supplementary Methods).

**Table 1.** Summary of Clinical Information Related to the Subjects. Subjects at Time0 Are all Cognitively Healthy and Grouped Into Stable Controls (CON_time0) or Future Converters to Dementia (DEM_time0)

Time Point	time0, *n* = 34			timeL, *n* = 34		
Group	Stable Control (CON_time0)	Dementia Converter (DEM_time0)	*p* Value	Stable Control (CON_timeL)	Dementia Converter (DEM_timeL)	*p* Value
Number of subjects	17	17		17	17	
Sex (M/F)	8/9	3/14	n.s.	8/9	3/14	n.s.
Age, mean yr (*SD*)	76.1 (2.8)	76.6 (4.1)	n.s.	80.1 (2.8)	80.6 (4.2)	n.s.
MMSE, mean (*SD*)	28.6 (1.5)	27.2 (2.4)	n.s.	28.5 (2.0)	21.2 (4.5)	***
FAQ, mean (*SD*)	0.4 (0.5)	0.9 (1.1)	n.s.	0.5 (0.8)	13.5 (8.5)	***
GDS, mean (*SD*)	1.2 (1.4)	1.8 (1.5)	n.s.	1.4 (2.0)	2.9 (2.0)	*
CDR, mean (*SD*)	0.0 (0.0)	0.0 (0.1)	n.s.	0.0 (0.1)	1.1 (0.3)	***

*Notes:* CDR = Clinical Dementia Rating; FAQ = Functional Activities Questionnaire; GDS = Geriatric Depression Scale; MMSE = Mini-Mental State Examination; *SD* = Standard Deviation. The same subjects are evaluated at timeL, when stable controls remain cognitively healthy (CON_timeL) while converters manifest the disease (DEM_timeL).

n.s., *p* ≥ .05, *p < .05, ***p < .001 for Wilcoxon rank sum or chi-squared tests.

## Results

We used empirical Bayes modified *t* tests in a linear model framework (11) to define differentially methylated probes of CpG sites (DMPs; false discovery rate [FDR] < 0.05) across the different comparisons. The models were adjusted to account for experimental processing batch, blood cell-type composition, sex, and subject-specific effects, with cell-type composition being predicted from the DNA methylation data using the Houseman algorithm (12) (Supplementary Methods). We employed variance decomposition methods to determine the potential effects of experimental or technical variables in our data. A surrogate variable analysis (13) confirmed that the 2 main variables driving latent variation in our data were batch and cell-type composition (principally CD8-T cells; Supplementary Figure 1). After carrying out the differential analyses, we found no statistically significant DMPs between control and converter individuals at either time0 or at timeL, while we did detect 14 DMPs in the longitudinal comparison (described in Supplementary Table 2). However, because the longitudinal comparison involves all subjects (34 at time0 vs 34 at timeL) while the dementia comparisons only involve half of the cohort (17 vs 17), the observed differences in detected DMPs could be due to an increase in statistical power. We performed subsampling of the cohort to retain only 17 individuals and repeated the longitudinal comparisons, finding no statistically significant DMPs across 5 iterations. These initial results suggest that, at the level of individual CpG sites, there are no marked DNA methylation alterations that are predictive of the development of dementia, or directly associated with this disease in the blood of older adults. We also found no evidence of an increase in DNA methylation differences between individuals after onset of symptoms in the DEM group as compared to differences at the prediagnosis stage.

To expand the biological exploration of our data, we next focused on the top probes for each comparison (unadjusted *p* < .05). These corresponded to 30 492 loci, 28 457 loci, and 35 007 loci, respectively, for the time0, timeL, and longitudinal comparisons (Figure 1b). These borderline CpG sites may collectively carry biological insight and, moreover, we validated 2 sites with moderate (*p* < .001, cg16593113) and marginal (*p* ~ .06, cg06937882) significance in the time0 and timeL comparisons by using bisulfite pyrosequencing in a subset of the samples (Figure 1c; Supplementary Table 3 for primer information), indicating that the array produced robust measurements. Indeed, the array and pyrosequencing measurements were highly concordant across all observations (Pearson correlation coefficient = 0.98, Supplementary Figure 2).

The time0-, timeL-, and longitudinal-DMPs were each associated with specific distributions across CpG island and gene locations (Figure 1d), with parallel hyper- or hypomethylation-specific trends being observed for all 3 comparisons. We analyzed the intersections between the sets of DMPs (Figure 1e, left plot) and found a strong enrichment in shared time0- and timeL-DMPs (Fisher’s test *p* < .001, odds ratio [OR] = 12). Moreover, the direction of the dementia-associated alterations was maintained at both time points (Figure 1e, right plot). When looking specifically at the DMPs common to both time0 and timeL that had a concordant direction of change (7 834 out of 7 875), we found no evidence of an increase in the magnitude of change at postdiagnosis (timeL) with respect to prediagnosis (time0; Wilcoxon rank sum test *p* = .257; Supplementary Figure 3). These results suggest that blood dementia-associated DNA methylation patterns are very similar at the prediagnosis stage and after the onset of dementia symptoms, and that these loci are different from those associated with longitudinal drift. We also performed Gene Ontology enrichment analyses on the sets of DMPs (Supplementary Table 4 for full results). Looking at the specific pathways detected for each comparison, we observed common trends for the hypermethylation of neural development pathways associated with time0, timeL, and also longitudinal DMPs (Supplementary Figure 4), indicating that the discernible DNA methylation alterations occurring in dementia at the prediagnosis or diagnosis stage may be linked to specific, and similar, functional pathways.

**Figure 2.**
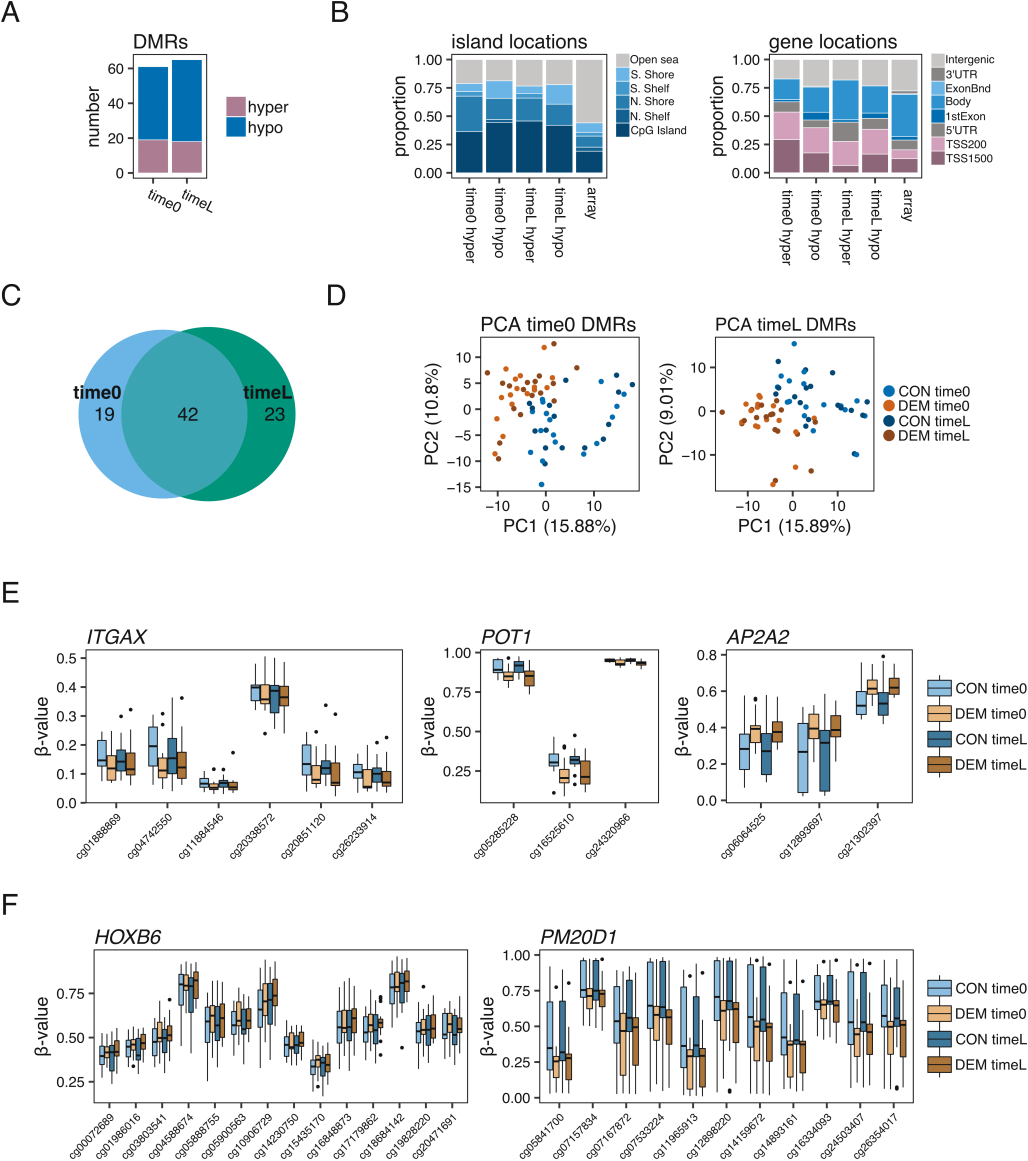
Regional DNA methylation alterations in dementia. (A) Barplots showing the numbers of hyper- and hypo-methylated dementia-associated DMRs (Sidak-adjusted *p* < .05) found in the time0 and timeL comparisons. (B) Barplots indicating the relative distribution of CpGs belonging to hyper- and hypo-methylated DMRs in the time0 and timeL comparisons, according to their CpG island location status (left) and gene location status (right). (C) Venn diagram showing the number of DMRs overlapping between the time0 and timeL comparisons. (D) Scatter plots describing the principal component analysis (PCA) of the study subjects according to their mean methylation values for the time0 or timeL DMRs. (E) Boxplots showing the measured DNA methylation values of the individuals, according to their experimental group, at the CpG sites belonging to DMRs associated with the *ITGAX*, *POT1,* and *AP2A2* genes. (F) Boxplots showing the measured DNA methylation values of the individuals, according to their experimental group, at the CpG sites belonging to 2 DMRs discovered in the integrative analysis associated with the *HOXB6* and *PM20D1* genes. CON = control; DEM = dementia; DMRs = differentially methylated regions.

Recent studies using larger cohorts (14,15) have failed to detect single-CpG biomarkers at an adequate significance level, while nonetheless being able to define differentially methylated regions (DMRs). Indeed, it is probable that the subtle DNA methylation alterations associated with dementia and cognitive decline in peripheral blood are better detected when looking at coordinated, region-level changes. Working along these lines, we performed a regional analysis to look for DMRs using the comb-p method (16) (Supplementary Methods). Interestingly, we detected 61 and 65 significant DMRs (Sidak-corrected *p* < .05; Supplementary Table 5 for lists of DMRs) between DEM and CON individuals at time0 and timeL, respectively, while detecting no significant regions for the longitudinal comparison, in spite of the latter comparison involving more subjects. These significant DMRs were dominated by hypomethylation changes (Figure 2a), and the CpGs involved were notably enriched at CpG islands and transcription start sites (TSS; Figure 2b; Fisher’s tests all *p* < .001, ORs = 2.4–3.6 for island and 1.5–4.5 for TSS enrichments, except for timeL hyper-DMRs which had a *p* value of .09 for TSS enrichment), indicating that they might have more defined roles as regards biological regulation. The majority of these regions (42) overlapped in time0 and timeL (Figure 2c), and all DMRs were altered in the same direction, indicating that the dementia-associated DMRs were already present in time0 individuals prior to the detection of overt cognitive decline symptoms. Indeed, the methylation status of the regions perfectly distinguished CON subjects from DEM subjects at both time points (Figure 2d). We did not, however, observe an increase in the magnitude of the alterations at these regions at timeL as opposed to time0 (Supplementary Figure 5).

In addition, because there is a gradient of cognitive scores within the dementia timeL subjects (Table 1; Supplementary Figure 6), the methylation levels at these regions could be subtly associated with the degree of cognitive decline. To explore this, we correlated neurological scores with mean DNA methylation values at the 42 overlapping DMRs by using linear models within the DEM timeL subgroup (Supplementary Methods), but no relationships were found to be significant after multiple-testing adjustment (FDR < 0.05).

A considerable proportion of the DMRs detected were mapped to genes functionally linked to Alzheimer’s or related pathologies via different mechanisms (Figure 2e; Supplementary Table 5) such as: (a) genes associated with polymorphisms related to Alzheimer’s disease risk—*PON1* (17), *AP2A2* (18), or *SH3PXD2A* (19) (although the latter is cohort-dependent (20))—or those associated with polymorphisms linked to Aβ-related neurodegeneration—*MAGI2* (21)—or polymorphisms related to cerebrospinal fluid tau phosphorylation levels—*POT1* (22); (b) genes with functional roles in dementia disease models—*ALOX5AP* (23), *PLK2* (24), or *ITGAX* (25); (c) tau protein-interacting genes—*PACSIN1* (26); (d) genes with plasma protein levels associated with Alzheimer’s in ApoE4 carriers—*CDH6* (27)—or upregulated in the peripheral blood of fast-progression subjects with early Alzheimer’s—*SLC2A8* (28); (e) genes associated with more general brain-pathology pathways—*CBR1* (29).

We next looked for overlaps between our study DMRs and those reported using external cohorts. First, we examined the regions with blood DNA methylation alterations in pre and postdiagnosis Alzheimer’s subjects described by Fransquet et al. (14) and found 13 intersections with our DMRs (overlapping or <1 000 bp in distance), 10 of which were altered in the same direction (Supplementary Table 6), including regions mapping to aforementioned genes such as *ALOX5AP*. We also found up to 18 intersections with the Alzheimer’s and MCI-associated blood altered regions reported by Wang et al. (30), 6 having the same direction of change (Supplementary Table 6), including, for example, a timeL DMR mapped to the *EIF4E* gene, a gene which has been recently reported as specifically detected in the lacrimal fluid of Alzheimer’s patients (31). Lastly, despite the fact that most of our regions were hypomethylated, and although it did not reach statistical significance in the DMR calling, we also confirmed in our cohort the recently reported hypermethylation of the *HOXB6* gene in the blood of Alzheimer’s patients (15). Interestingly, as is the case for most of our regions, we observed that the DNA methylation alterations were already present in our prediagnosis time0 samples. Taken together, these results highlight the importance of describing cohort-independent DNA methylation alterations. To further pursue this, we made use of the raw data shared by Roubroeks et al. (15) and performed a DMR analysis by integrating their data set with our own measurements (Supplementary Methods). With this strategy, we discovered 8 cohort-independent DMRs (Supplementary Table 7; Figure 2f), which included genes such as *HOXB6*, mentioned earlier, and also *PM20D1*, which has been recently described as hypomethylated in the peripheral blood of early Alzheimer’s (30) and is a quantitative trait locus in this disease (32).

Finally, we screened various epigenetic clocks in order to look for more general epigenomic alterations. We estimated DNAm ages using the “Hannum” blood DNAm clock (33), the “Horvath” universal DNAm clock (34), the “PhenoAge” DNAm clock (35), the “GrimAge” DNAm clock (36), and the “Telomere” DNAm clock (37). We computed DNAm age acceleration values by extracting the residuals from the regression of DNAm age on chronological age, with GrimAge also being adjusted for sex after we observed a significant association with this variable (Supplementary Methods). We found no significant differences in DNAm age acceleration or DNAm telomere length acceleration across the groups (Supplementary Figure 7). DNAm age acceleration has been repeatedly associated with Alzheimer phenotypes in brain tissue (35,38,39), while mixed results have been obtained in blood, with DNAm age acceleration having been linked to cognitive fitness, but not to its longitudinal decline (40), associated with longitudinal cognitive decline (41) or not associated with any differences whatsoever (42). In the case of our cohort, there did not appear to be noticeable differences in epigenetic age acceleration between the groups studied. To take into account the distribution of cognitive scores within the dementia timeL subjects (Table 1; Supplementary Figure 6), we also correlated the acceleration with neurological score values by using linear models across all subjects and also within the dementia timeL subgroup. We again found little evidence of a robust increase in epigenetic age acceleration linked to cognitive decline across the 5 different epigenetic clocks, and no significant association was observed after adjustment for multiple testing (Supplementary Figures 8 and 9).

## Discussion

In summary, our work describes DNA methylation alterations in the peripheral blood mononuclear cells of cognitively healthy older adults who in the medium-term (at 4-year follow-up) either develop dementia or remain cognitively healthy. Importantly, most of the observed alterations are present at both the prediagnosis and the postdiagnosis stage, suggesting that DNA methylation alterations associated with dementia have already accumulated in peripheral tissues such as blood prior to clinical symptoms being observed, thus indicating its value for the development of epigenetic biomarkers of disease. Even so, these observations could perhaps also be explained by the presence of preexisting individual genetic traits. While it is true that DNA methylation alterations are better detected when looking at coordinated, regional changes, the exploration of the DNA methylation patterns at the single-CpG level also reveals distinctive signatures associated with biological features.

Our study provides valuable epigenetic profiling, using Illumina MethylationEPIC arrays, of a well-characterized longitudinal cohort with comprehensive cognitive measurements separated by a 4-year span. This design allowed for comparisons to be made at 2-time points and also longitudinally. On the other hand, the main limitations of the investigation were: (a) the sample size, which likely limited the power to detect more subtle alterations; (b) the use of a general diagnosis of dementia without differentiating specific subtypes, so that the alterations described here could be related to other pathologies such as vascular dementia; (c) the short time span in terms of detecting robust aging alterations; and (d) the lack of analyses of lifestyle variables which could reveal additional insights on the biological processes involved.

The DMRs described in this study are related to many Alzheimer’s-associated genes, and also overlap with regions reported in other studies, indicating that epigenetic changes reflect the underlying biological processes at play in the development of this disease. Nonetheless, the numbers and extent of dementia-associated DNA methylation alterations are limited, and as such, there is a need for high-powered studies which facilitate the detection of more subtle, poly-epigenetic traits. In this sense, the public availability of epigenetic profiling data sets is of great value for the integration and validation of future studies.

## Supplementary Material

glac068_suppl_Supplementary_Material

glac068_suppl_Supplementary_Table_S1

glac068_suppl_Supplementary_Table_S2

glac068_suppl_Supplementary_Table_S3

glac068_suppl_Supplementary_Table_S4

glac068_suppl_Supplementary_Table_S5

glac068_suppl_Supplementary_Table_S6

glac068_suppl_Supplementary_Table_S7

## Data Availability

All data generated during this study are included in this published article and its Supplementary Information Files. The raw IDAT and preprocessed data are also available in the ArrayExpress public repository under accession E-MTAB-10600.
