# Supplementary material for: Blood DNA Methylation Patterns in Older Adults With Evolving Dementia
Source: J Gerontol A Biol Sci Med Sci. 2022 Mar 17;77(9):1743–9. doi: 10.1093/gerona/glac068 (PMC9434456; doi:10.1093/gerona/glac068)
Supplement: glac068_suppl_Supplementary_Material [file glac068_suppl_supplementary_material.pdf]

ONLINE-MATERIAL SUPPORTING INFORMATION

- Supplementary eMethods
- Supplementary eTables 1-7
- Supplementary eFigures 1-9

The raw IDAT and pre-processed data are also available in the ArrayExpress public repository under accession E-MTAB-10600.

eMETHODS

Study subjects

The subjects involved in this study were volunteers participating in "The Vallecas Project" cohort, a single-center longitudinal community-based study (1). All subjects were genotyped for ApoE4 status. 68 samples from a total of 34 subjects were analysed, consisting of 17 control (CON) and 17 converter individuals (DEM) evaluated at two time points: pre-diagnosis (time0) and post-diagnosis (timeL). Control individuals remained cognitively healthy at both time points, while converter individuals were cognitively healthy at time0 and had been diagnosed with dementia by timeL (see below). The groups were labelled as CON_time0, CON_timeL, DEM_time0 and DEM_timeL; see Table 1 and eTable 1 for additional information.

Yearly evaluations and cognitive assessment

The study subjects were evaluated annually on a series of physiological and neurological parameters. On the baseline visit, sociodemographic data, vital signs, and blood samples were collected, and followed by neuropsychological, clinical and multi-sequence MRI assessment. The same procedure was repeated in subsequent yearly follow-up visits, excluding the genetic testing.

Neuropsychological testing consisted of a comprehensive battery of tests. Cognitive performance: Mini-Mental State Examination (MMSE), Free and Cued Selective Reminding Test (FCSRT) and Rey–Osterrieth Complex Figure (only on baseline visit) as well as phonological and semantic verbal fluency; Depression and Anxiety: Geriatric Depression Scale (GDS) and State-Trait Anxiety Inventory (STAI); Functional scales: Clinical Dementia Rating (CDR), Functional Activities Questionnaire (FAQ). A more detailed description of the design, demographic and neuropsychological assessment and clinical evaluation of the "Vallecas Project" is available elsewhere (1). Diagnosis of dementia was made following Petersen's criteria (2) and NIA-AA recommendations (3). Participants who had developed mild dementia at the follow-up visits were considered "converters" and those who remained cognitively healthy were considered "controls". The diagnosis of dementia was agreed between 2 experienced clinicians, one a neurologist, the other a neuropsychologist. In the case of lack of agreement between the neurologist and neuropsychologist about the diagnosis of any particular individual, the case was reviewed at an independent consensus meeting involving 3 further members of the research team (neurologists and neuropsychologists).

DNA extraction and methylation analysis

DNA from peripheral blood mononuclear cells was extracted following a standard phenol-chloroform protocol. Next, the DNA was bisulfite converted using the EZ-96 DNA Methylation Kit conversion protocol (Zymo Research). Finally, the Illumina Infinium HD Methylation Assay protocol was performed, hybridising samples to Infinium MethylationEPIC BeadChips.

Array data pre-processing

Data pre-processing and analysis were carried out with the statistical software R (v3.6.2). IDAT files were imported and pre-processed using the *minfi* package (v1.32.0) (4). Background correction was performed with the ssNoob method (5) and normalization of extracted β-values by the BMIQ method (6). Self-reported sex and subject genetic tracking were validated by accessing the array methylation data for sex chromosome probes and SNP probes, using the *getSex* and *getSnpBeta* functions from *minfi*.

Finally, probes mapping to sex chromosomes, probes with detection p-values >0.01 in any sample, probes not included in the Illumina MethylationEPIC B4 annotation, cross-reactive and multimapping probes (7,8) and probes with SNPs with MAF ≥0.01 at their CpG or SBE sites (dbSNP v147) were filtered out. In addition, experiment-specific conflicting probes identified by clustered-distribution analysis using the *gaphunter* function of the *minfi* package (threshold = 0.25, outCutoff = 3/68) were also removed (9). The final number of probes analysed was 771,473.

Analyses of differentially methylated CpGs

Statistical analyses were performed by, first, logit-transforming β-values to M-values. The detection of differentially methylated probes (DMPs) was performed using the *limma* package (v3.42.2) (10). Linear models were built fitting M-values as dependent variable and group (CON_time0, CON_timeL, DEM_time0, DEM_timeL) as independent variable. Comparisons were carried out at the pre-diagnosis time point (time0: CON_time0 vs DEM_time0), at the post-diagnosis time point (timeL: CON_timeL vs DEM_timeL) and also to derive longitudinal changes (longitudinal: CON_time0 ∪ DEM_time0 vs CON_timeL ∪ DEM_timeL). DMPs were defined by contrasting coefficients using an empirical Bayes moderated *t-test.* Initially, those with a Benjamini & Hochberg (FDR) adjusted of p<0.05 were classified as statistically significant. Further analyses were also performed for probes with unadjusted p<0.05.

All of the models were adjusted for experimental processing batch, cell-type composition, sex and subject-specific effects, the latter being accounted for through a random effect component. A surrogate variable analysis (SVA) was applied using the *sva* package (v3.34.0, "leek" method) (11), and showed that batch and T-lymphocyte levels were the main latent variables impacting our dataset (see eFigure 1).

Analyses of differentially methylated regions

Differentially methylated regions (DMRs) were detected using the "comb-p" method (12) implemented in the *ENmix* package (v1.28.2) (13) under default parameters and FDR<0.05. In brief, p-values obtained from the *limma* DMP analyses were used as input in order to find spatially-correlated regions of significance, which are first selected under an initial FDR threshold. Then, significant DMRs were defined as those with a Sidak-corrected p<0.05, a change of at least 1% in mean methylation values between the compared groups and containing at least 3 CpG sites.

To correlate DMRs with cognitive scores, mean methylation values were computed for each DMR and subsequently transformed to M-values. Then, using the *limma* package (v3.42.2), linear models were built to find associations between the mean methylation values at the DMRs and cognitive score levels, while adjusting for covariates (batch, sex, cell-type composition, subject). Cognitive scores were treated as numerical variables.

Probe and region annotation and testing

The *IlluminaHumanMethylationEPICanno.ilm10b4.hg19* package (v0.6.0) was used to annotate array probes to genomic locations and genes. Fisher's exact tests were used to compare proportions of annotations and enrichments of intersections.

For the annotation of regions, the following strategy was applied: First, single CpGs belonging to each region were first individually annotated. Next, the regions were assigned a single annotation by selecting the annotations of their single CpGs with the following priorities: "Island">"N_Shore">"S_Shore">"N_Shelf">"S_Shelf">"OpenSea" for "Island" annotations and "TSS1500">"TSS200">"5'UTR">"1stExon">"Body">"ExonBnd">"3'UTR">"Intergenic" for "Gene" annotations.

Evaluation of overlaps between genomic loci was performed using the *GenomicRanges* package (v1.39.3) (14). DMRs were reported to overlap with regions reported in other studies (15,16) when directly intersecting or being adjacent (<1000 bp).

Cell-type composition prediction

Cell-type composition was predicted from the DNA methylation data using the Houseman algorithm (17) implemented in the *ENmix* package (v1.28.2) (13). Specific blood cell reference datasets available for the Illumina MethylationEPIC platform were employed for the estimation (18).

DNA methylation age computation

Epigenetic age was estimated for various DNA methylation clocks: the "Hannum" blood DNAm clock (19), the "Horvath" universal DNAm clock (20), the "PhenoAge" DNAm clock (21), the "GrimAge" DNAm clock (22) and the "Telomere" DNAm clock (23). Predicted DNAm ages and DNAm telomere lengths were obtained using the DNA Methylation Age Calculator tool with the normalization option selected (http://dnamage.genetics.ucla.edu/, accessed May 2021). Age acceleration and DNAm telomere length acceleration were defined as the residuals of adjusting the raw DNAm age values for chronological age in a linear model. GrimAge was also adjusted for sex after a significant association with this variable was observed.

Pathway enrichment analyses

Pathway enrichment analyses were performed with the *missMethyl* package (v1.20.4) (24) using the *gometh* function and using as background the filtered list of assayed probes. This method takes into account differences in the number of probes mapping to each gene. The Gene Ontology (GO) database was interrogated. When representing the results (see Figure S4), large, uninformative gene sets (>1500 genes) were removed.

Bisulfite pyrosequencing

The DNA methylation levels of 2 representative CpG sites (cg16593113, cg06937882) were estimated by bisulfite pyrosequencing (see eTable 3 for primer information) in a subset (n = 24) of the study samples. Extracted DNA was bisulfite converted with the EZ-96 DNA Methylation-Gold Kit (Zymo research), amplified by PCR with biotinylated primers and pyrosequenced using PyroMark Q24 reagents and a vacuum prep workstation, equipment and software (Qiagen).

Integration of external datasets and differential methylation analyses

To perform integrative analyses, dasen-normalized β-values from Infinium HumanMethylation450K arrays of whole blood control and Alzheimer's samples from (25) were accessed via Gene Expression Omnibus (accession number GSE144858) (26). These measurements were integrated with our study's Infinium MethylationEPIC data from timeL subjects (CON_timeL, DEM_timeL), to retain a final 367,365 measured probes in both cohorts.

The differential methylation analyses were performed as described previously: Firstly, the *limma* package was used to perform comparisons at the single-CpG level between Alzheimer's cases and controls; secondly, the p-values obtained

from the *limma* DMP analyses were analysed via the “comb-p” method to define DMRs, which were required to have a Sidak-corrected $p<0.05$, a change of at least 1% in mean methylation values between the groups compared (for both cohorts independently) and to contain at least 3 CpG sites. The statistical models used were adjusted for sex, study batch, and cell-type compositions. The cell-type compositions for the GSE144858 data were predicted using *ENmix* as mentioned above, using datasets specific to the 450K platform (27).

Other analyses

Principal component analysis (PCA) was performed using the R package *stats* (v3.6.2), and categorical PCA (CATPCA) was computed using the R package *Gifi* (v0.3.9) (https://cran.r-project.org/web/packages/Gifi/index.html).

eMETHODS REFERENCES

1. Olazarán J, Valentí M, Frades B, et al. The Vallecas Project: A Cohort to Identify Early Markers and Mechanisms of Alzheimer's Disease. *Front Aging Neurosci*. 2015;7:181. doi:10.3389/fnagi.2015.00181.

2. Petersen RC. Mild cognitive impairment as a diagnostic entity. *J Intern Med*. 2004;256(3):183-194. doi:10.1111/j.1365-2796.2004.01388.x.

3. Albert MS, DeKosky ST, Dickson D, et al. The diagnosis of mild cognitive impairment due to Alzheimer's disease: Recommendations from the National Institute on Aging-Alzheimer's Association workgroups on diagnostic guidelines for Alzheimer's disease. *Alzheimers Dement*. 2011;7(3):270-279. doi:10.1016/j.jalz.2011.03.008.

4. Aryee MJ, Jaffe AE, Corrada-Bravo H, et al. Minfi: a flexible and comprehensive Bioconductor package for the analysis of Infinium DNA methylation microarrays. *Bioinformatics*. 2014;30(10):1363-1369. doi:10.1093/bioinformatics/btu049.

5. Triche TJ, Weisenberger DJ, Van Den Berg D, Laird PW, Siegmund KD. Low-level processing of Illumina Infinium DNA Methylation BeadArrays. *Nucleic Acids Research*. 2013;41(7):e90-e90. doi:10.1093/nar/gkt090.

6. Teschendorff AE, Marabita F, Lechner M, et al. A beta-mixture quantile normalization method for correcting probe design bias in Illumina Infinium 450 k DNA methylation data. *Bioinformatics*. 2013;29(2):189-196. doi:10.1093/bioinformatics/bts680.

7. Chen Y, Lemire M, Choufani S, et al. Discovery of cross-reactive probes and polymorphic CpGs in the Illumina Infinium HumanMethylation450 microarray. *Epigenetics*. 2013;8(2):203-209. doi:10.4161/epi.23470.

8. Pidsley R, Zotenko E, Peters TJ, et al. Critical evaluation of the Illumina MethylationEPIC BeadChip microarray for whole-genome DNA methylation profiling. *Genome Biol*. 2016;17(1):208. doi:10.1186/s13059-016-1066-1.

9. Andrews SV, Ladd-Acosta C, Feinberg AP, Hansen KD, Fallin MD. "Gap hunting" to characterize clustered probe signals in Illumina methylation array data. *Epigenetics Chromatin*. 2016;9:56. doi:10.1186/s13072-016-0107-z.

10. Ritchie ME, Phipson B, Wu D, et al. limma powers differential expression analyses for RNA-sequencing and microarray studies. *Nucleic Acids Research*. 2015;43(7):e47-e47. doi:10.1093/nar/gkv007.

11. Leek JT, Johnson WE, Parker HS, Jaffe AE, Storey JD. The sva package for removing batch effects and other unwanted variation in high-throughput experiments. *Bioinformatics*. 2012;28(6):882-883. doi:10.1093/bioinformatics/bts034.

12. Pedersen BS, Schwartz DA, Yang IV, Kechris KJ. Comb-p: software for combining, analyzing, grouping and correcting spatially correlated P-values. *Bioinformatics*. 2012;28(22):2986-2988. doi:10.1093/bioinformatics/bts545.

13. Xu Z, Niu L, Li L, Taylor JA. ENmix: a novel background correction method for Illumina HumanMethylation450 BeadChip. *Nucleic Acids Res*. 2016;44(3):e20. doi:10.1093/nar/gkv907.

14. Lawrence M, Huber W, Pagès H, et al. Software for computing and annotating genomic ranges. *PLoS Comput Biol*. 2013;9(8):e1003118. doi:10.1371/journal.pcbi.1003118.

15. Fransquet PD, Lacaze P, Saffery R, et al. Blood DNA methylation signatures to detect dementia prior to overt clinical symptoms. *Alzheimers Dement (Amst)*. 2020;12(1):e12056. doi:10.1002/dad2.12056.

16. Wang Q, Chen Y, Readhead B, et al. Longitudinal data in peripheral blood confirm that PM20D1 is a quantitative trait locus (QTL) for Alzheimer's disease and implicate its dynamic role in disease progression. *Clin Epigenetics*. 2020;12(1):189. doi:10.1186/s13148-020-00984-5.

17. Houseman EA, Accomando WP, Koestler DC, et al. DNA methylation arrays as surrogate measures of cell mixture distribution. *BMC Bioinformatics*. 2012;13:86. doi:10.1186/1471-2105-13-86.

18. Salas LA, Koestler DC, Butler RA, et al. An optimized library for reference-based deconvolution of whole-blood biospecimens assayed using the Illumina HumanMethylationEPIC BeadArray. *Genome Biol*. 2018;19(1):64. doi:10.1186/s13059-018-1448-7.

19. Hannum G, Guinney J, Zhao L, et al. Genome-wide methylation profiles reveal quantitative views of human aging rates. *Mol Cell*. 2013;49(2):359-367. doi:10.1016/j.molcel.2012.10.016.

20. Horvath S. DNA methylation age of human tissues and cell types. *Genome Biology*. 2013;14(10):R115. doi:10.1186/gb-2013-14-10-r115.

21. Levine ME, Lu AT, Quach A, et al. An epigenetic biomarker of aging for lifespan and healthspan. *Aging (Albany NY)*. 2018;10(4):573-591. doi:10.18632/aging.101414.

22. Lu AT, Quach A, Wilson JG, et al. DNA methylation GrimAge strongly predicts lifespan and healthspan. *aging*. 2019;11(2):303-327. doi:10.18632/aging.101684.

23. Lu AT, Seeboth A, Tsai P-C, et al. DNA methylation-based estimator of telomere length. *Aging (Albany NY)*. 2019;11(16):5895-5923. doi:10.18632/aging.102173.

24. Phipson B, Maksimovic J, Oshlack A. missMethyl: an R package for analyzing data from Illumina's HumanMethylation450 platform. *Bioinformatics*. 2016;32(2):286-288. doi:10.1093/bioinformatics/btv560.

25. Roubroeks JAY, Smith AR, Smith RG, et al. An epigenome-wide association study of Alzheimer's disease blood highlights robust DNA hypermethylation in the HOXB6 gene. *Neurobiol Aging*. 2020;95:26-45. doi:10.1016/j.neurobiolaging.2020.06.023.

26. Barrett T, Wilhite SE, Ledoux P, et al. NCBI GEO: archive for functional genomics data sets--update. *Nucleic Acids Res*. 2013;41(Database issue):D991-995. doi:10.1093/nar/gks1193.

27. Reinius LE, Acevedo N, Joerink M, et al. Differential DNA methylation in purified human blood cells: implications for cell lineage and studies on disease susceptibility. *PLoS One*. 2012;7(7):e41361. doi:10.1371/journal.pone.0041361.

eTABLE LEGENDS

eTable 1. Expanded clinical information related to the subjects.

eTable 2. List and details of the 14 DMPs (FDR<0.05) found in the longitudinal comparison (time0 vs timeL).

eTable 3. Primer sequences for bisulfite sequencing of the validated CpGs.

eTable 4. Full information on the enrichment of different gene ontology sets in the hyper- and hypomethylated DMPs.

eTable 5. Lists and details of the DMRs (Sidak-corrected p<0.05) found between DEM and CON subjects at time0 and timeL.

eTable 6. Lists and details of the DMRs (Sidak-corrected p<0.05) found to overlap with regions described in other studies.

eTable 7. Lists of the DMRs (Sidak-corrected p<0.05) between DEM and CON subjects by integrating the study cohort and the cohort described in GSE144858.

eFIGURES

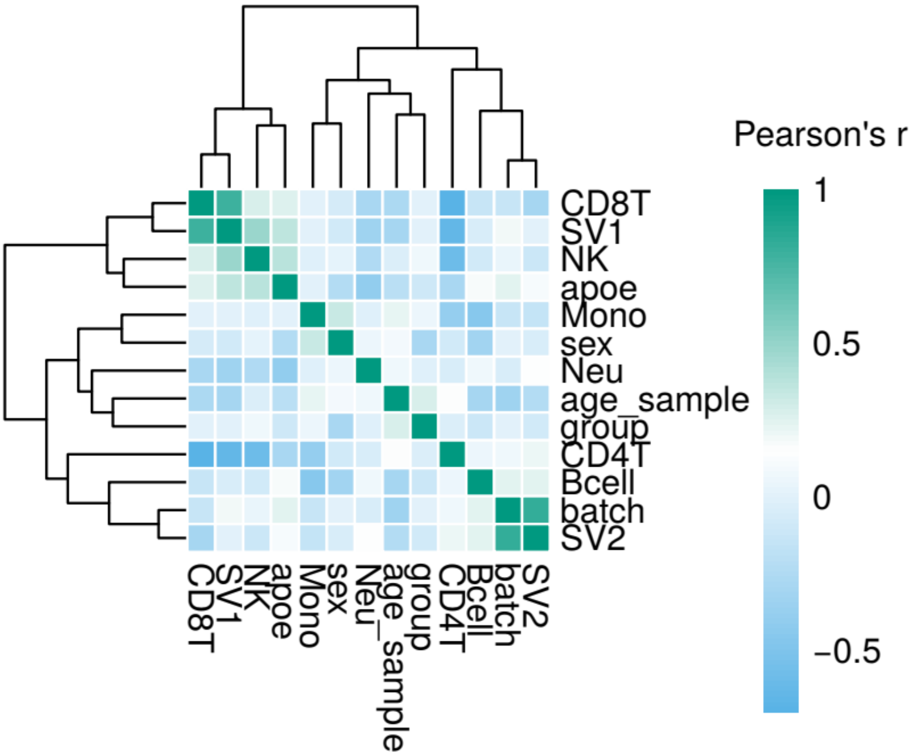

eFigure 1. Heatmap describing the correlations (Pearson's r) between clinical variables and latent surrogate variables (SV1, SV2) extracted from the data. Categorical variables were transformed into numerical variables to compute the correlations.

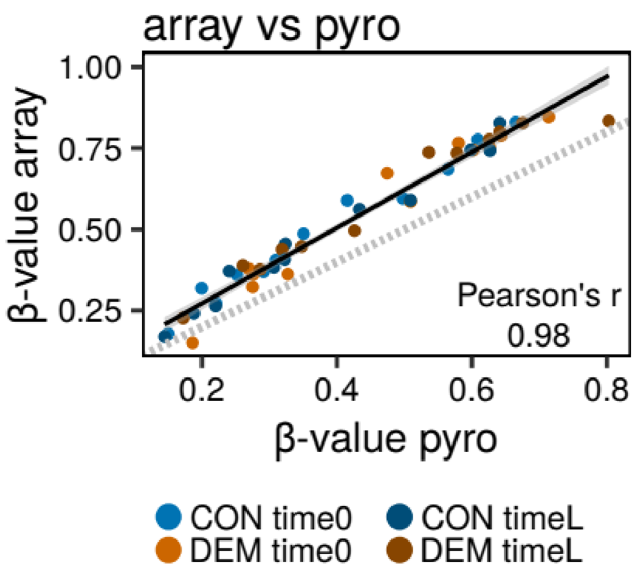

eFigure 2. Scatter plot showing the correlation between DNA methylation measurements performed by the Infinium MethylationEPIC BeadChip and by bisulfite pyrosequencing for 2 CpGs (cg16593113, cg06937882) in a subset of 24 samples. The black line indicates the linear correlation estimate and the grey line indicates a 1:1 correspondence. The different colours represent different experimental groups, and the Pearson correlation coefficient is shown.

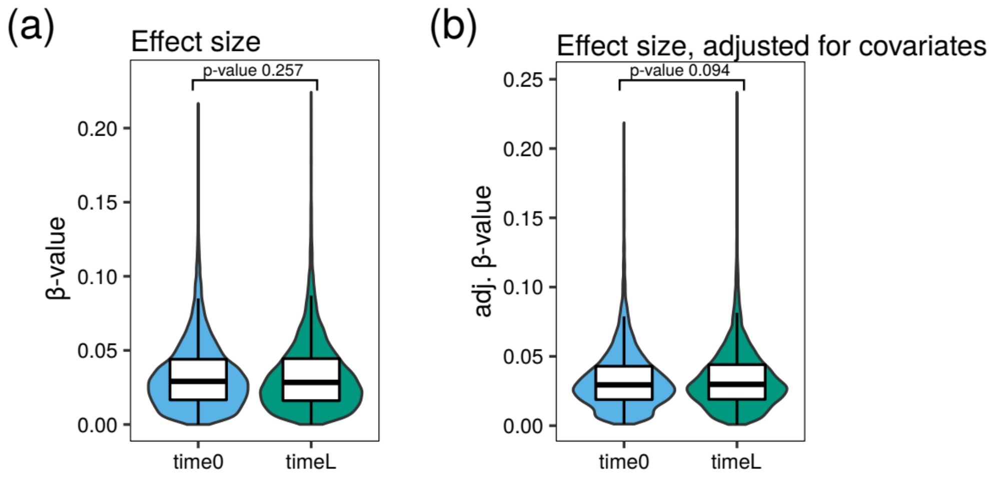

eFigure 3. (a) Violin plots describing the magnitude of change of common DMPs detected at time0 and timeL which had the same direction of change at both time points. The magnitude of change is measured as the absolute β-value difference between the mean methylation of each CpG in the CON versus the DEM group. The p-value from a Wilcoxon rank sum test is shown. (b) The same comparison is shown using covariate-adjusted β-values.

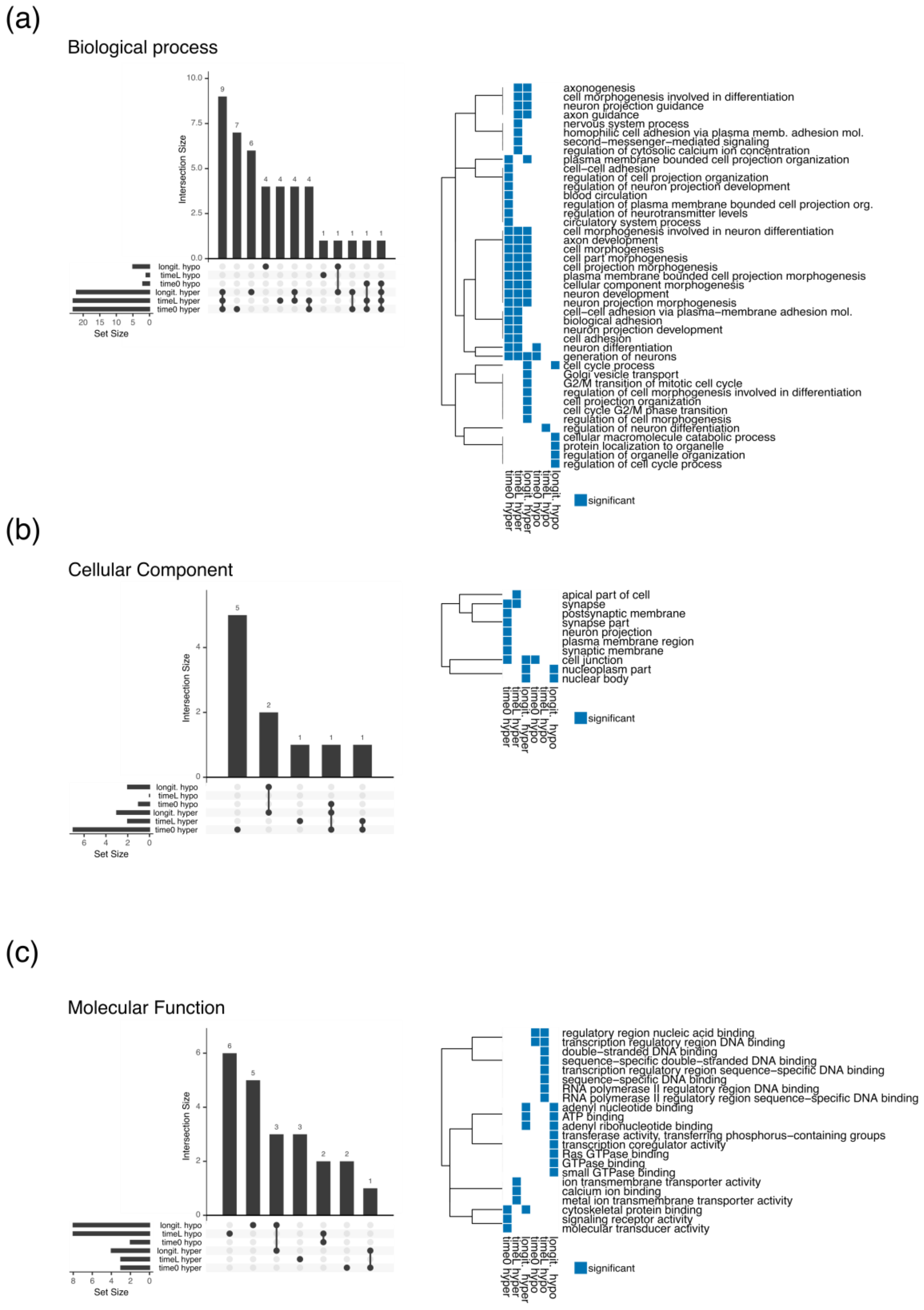
(a)
Biological process
Intersection Size
Set Size
longit. hypo
timeL hypo
time0 hypo
longit. hyper
timeL hyper
time0 hyper
axonogenesis
cell morphogenesis involved in differentiation
neuron projection guidance
axon guidance
nervous system process
homophilic cell adhesion via plasma memb. adhesion mol.
second−messenger−mediated signaling
regulation of cytosolic calcium ion concentration
plasma membrane bounded cell projection organization
cell−cell adhesion
regulation of cell projection organization
regulation of neuron projection development
blood circulation
regulation of plasma membrane bounded cell projection org.
regulation of neurotransmitter levels
circulatory system process
cell morphogenesis involved in neuron differentiation
axon development
cell morphogenesis
cell part morphogenesis
cell projection morphogenesis
plasma membrane bounded cell projection morphogenesis
cellular component morphogenesis
neuron development
neuron projection morphogenesis
cell−cell adhesion via plasma−membrane adhesion mol.
biological adhesion
neuron projection development
cell adhesion
neuron differentiation
generation of neurons
cell cycle process
Golgi vesicle transport
G2/M transition of mitotic cell cycle
regulation of cell morphogenesis involved in differentiation
cell projection organization
cell cycle G2/M phase transition
regulation of cell morphogenesis
regulation of neuron differentiation
cellular macromolecule catabolic process
protein localization to organelle
regulation of organelle organization
regulation of cell cycle process
significant
(b)
Cellular Component
Intersection Size
Set Size
apical part of cell
synapse
postsynaptic membrane
synapse part
neuron projection
plasma membrane region
synaptic membrane
cell junction
nucleoplasm part
nuclear body
significant
(c)
Molecular Function
Intersection Size
Set Size
regulatory region nucleic acid binding
transcription regulatory region DNA binding
double−stranded DNA binding
sequence−specific double−stranded DNA binding
transcription regulatory region sequence−specific DNA binding
sequence−specific DNA binding
RNA polymerase II regulatory region DNA binding
RNA polymerase II regulatory region sequence−specific DNA binding
adenyl nucleotide binding
ATP binding
adenyl ribonucleotide binding
transferase activity, transferring phosphorus−containing groups
transcription coregulator activity
Ras GTPase binding
GTPase binding
small GTPase binding
ion transmembrane transporter activity
calcium ion binding
metal ion transmembrane transporter activity
cytoskeletal protein binding
signaling receptor activity
molecular transducer activity
significant

eFigure 4. Heatmaps indicating the significantly enriched (FDR<0.01) gene ontology sets detected for hyper- and hypomethylated DMPs accompanied by UpSet plots indicating the intersections between the significant gene ontology sets for (a) "Biological process", (b) "Cellular Component" and (c) "Molecular Function" categories. Uninformative sets with N > 1500 genes are filtered out of the results.

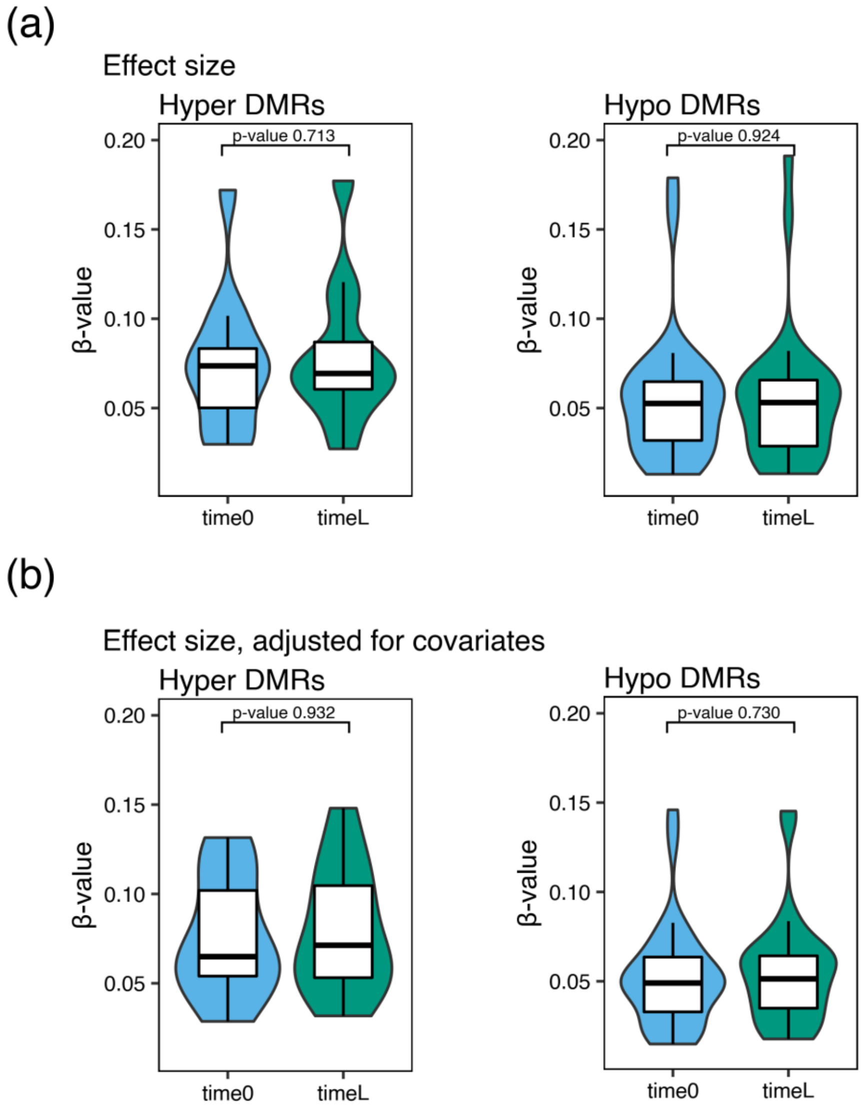

eFigure 5. (a) Violin plots indicating the magnitude of change of CpG sites belonging to the 42 overlapping DMRs detected at time0 and timeL. The magnitude of change is measured as the absolute β-value difference between the mean methylation of each CpG in the CON versus the DEM group. The p-value from a Wilcoxon rank sum test is shown. (b) The same comparison shown using covariate-adjusted β-values.

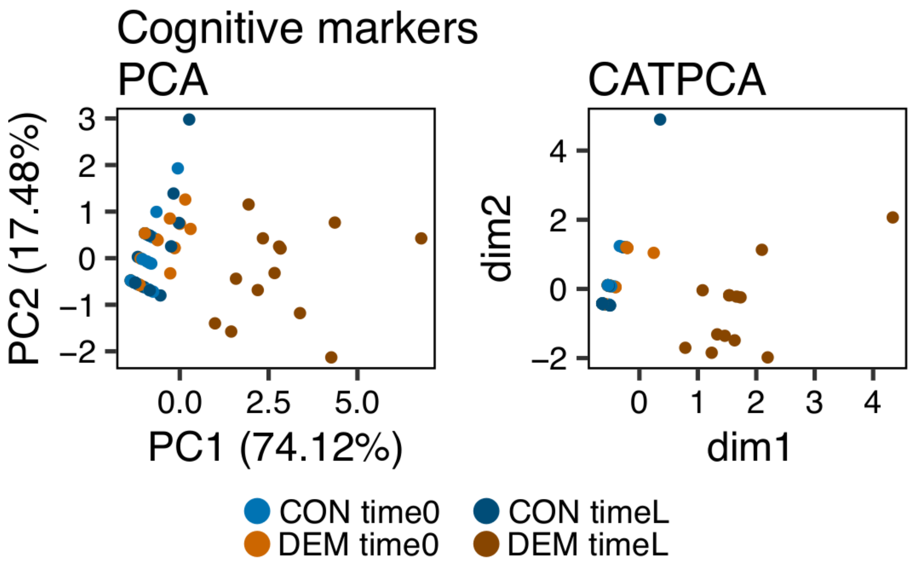

eFigure 6. Scatter plots showing the principal component analysis (PCA, left) or categorical PCA (CATPCA, right) of the study subjects according to their cognitive scores across different cognitive markers (MMSE, FAQ, GDS, CDR). The analyses were performed for subjects with complete measurements across all variables. MMSE: Mini Mental State Examination; FAQ: Functional Activities Questionnaire; GDS: Geriatric Depression Scale; CDR: Clinical Dementia Rating.

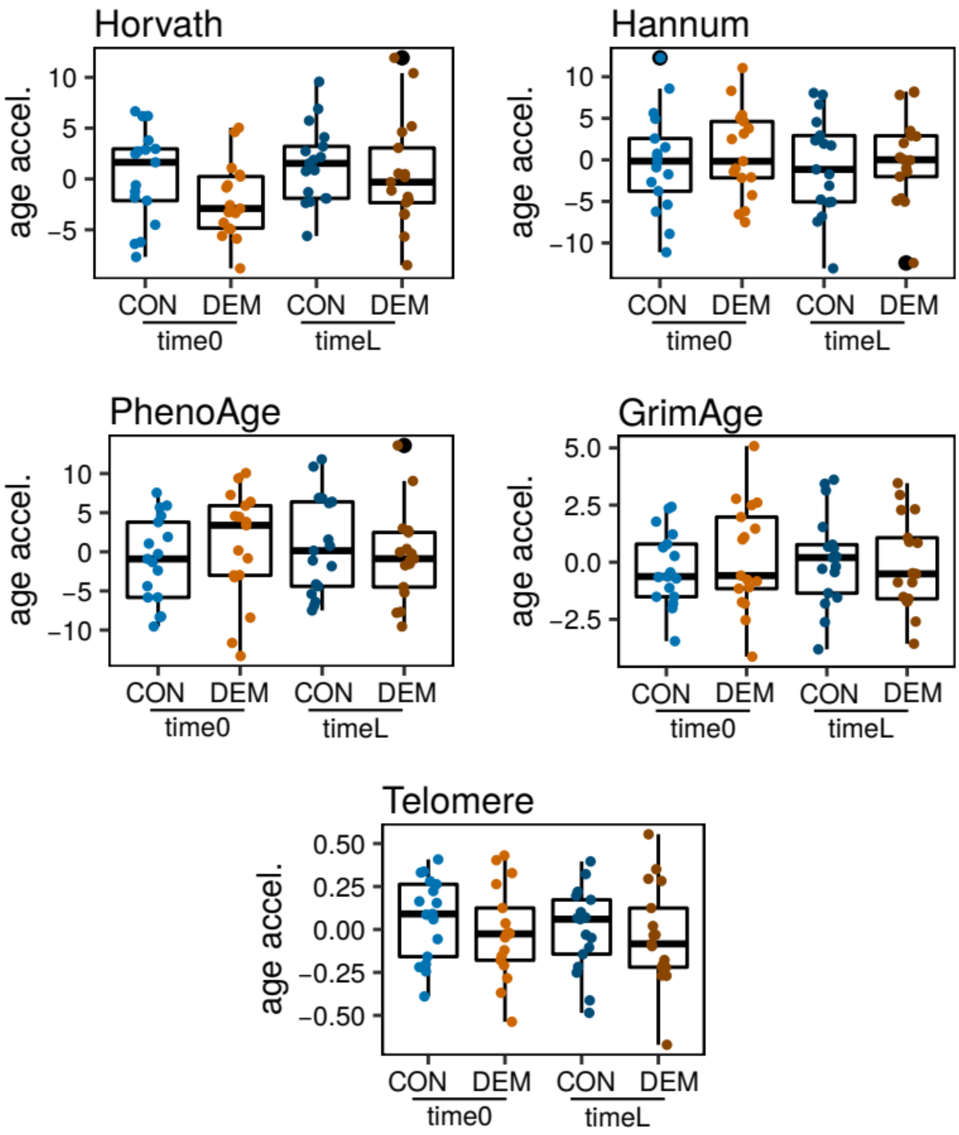

eFigure 7. Boxplots showing age acceleration values across the different study groups for different DNA methylation clocks. Age acceleration values correspond to the residuals of adjusting predicted DNAm ages for chronological age. In the case of GrimAge, the DNAm ages are also adjusted for sex. There were no significant differences between any group comparison (t-tests for time0, timeL or age comparisons).

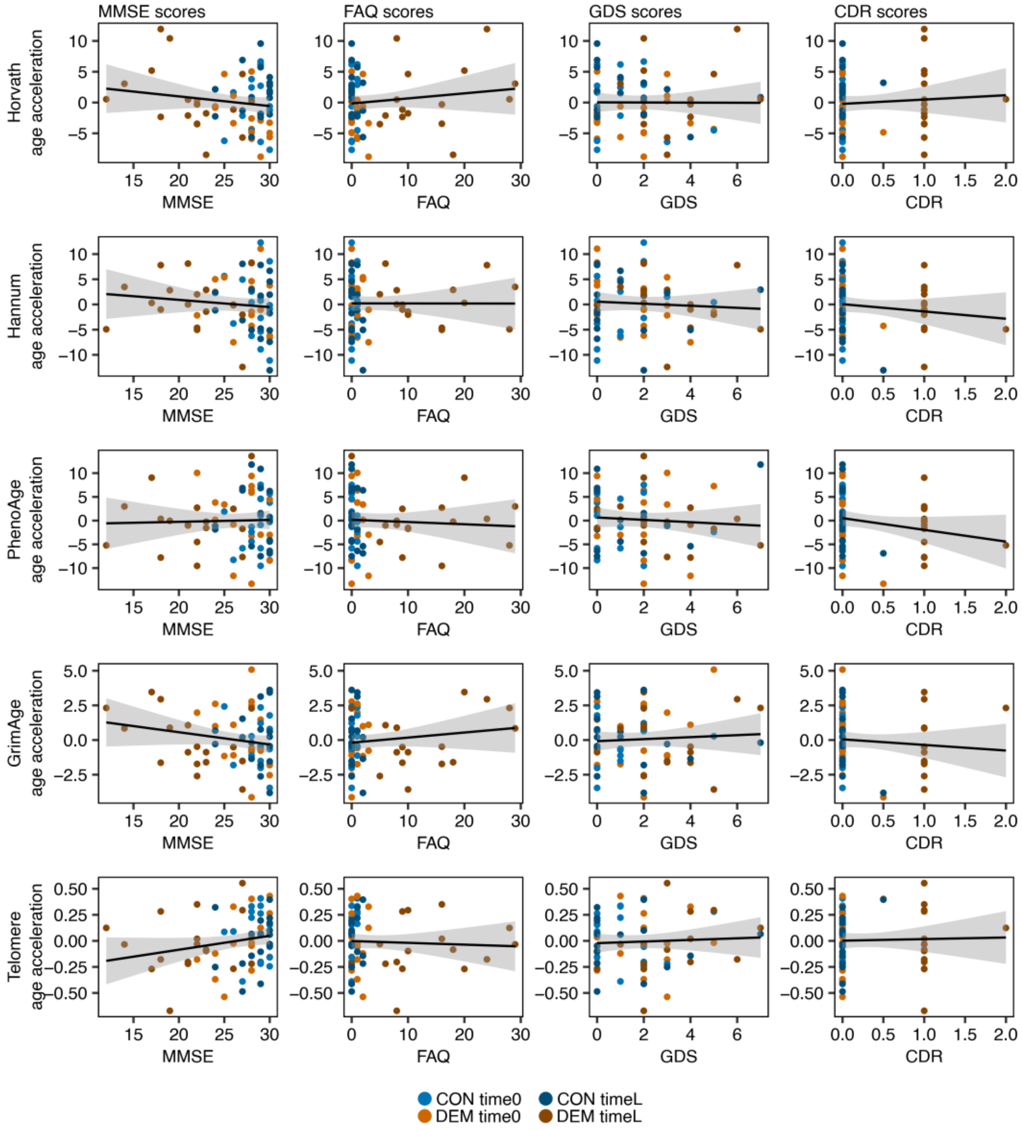

eFigure 8. Scatter plots showing the correlation between epigenetic age acceleration measurements for different epigenetic clocks (Horvath, Hannum, PhenoAge, GrimAge, Telomere) and neurological markers (MMSE, FAQ, GDS, CDR) across the study subjects. The black lines indicate linear fit. No significant associations were observed after adjustment for multiple testing. MMSE: Mini Mental State Examination; FAQ: Functional Activities Questionnaire; GDS: Geriatric Depression Scale; CDR: Clinical Dementia Rating.

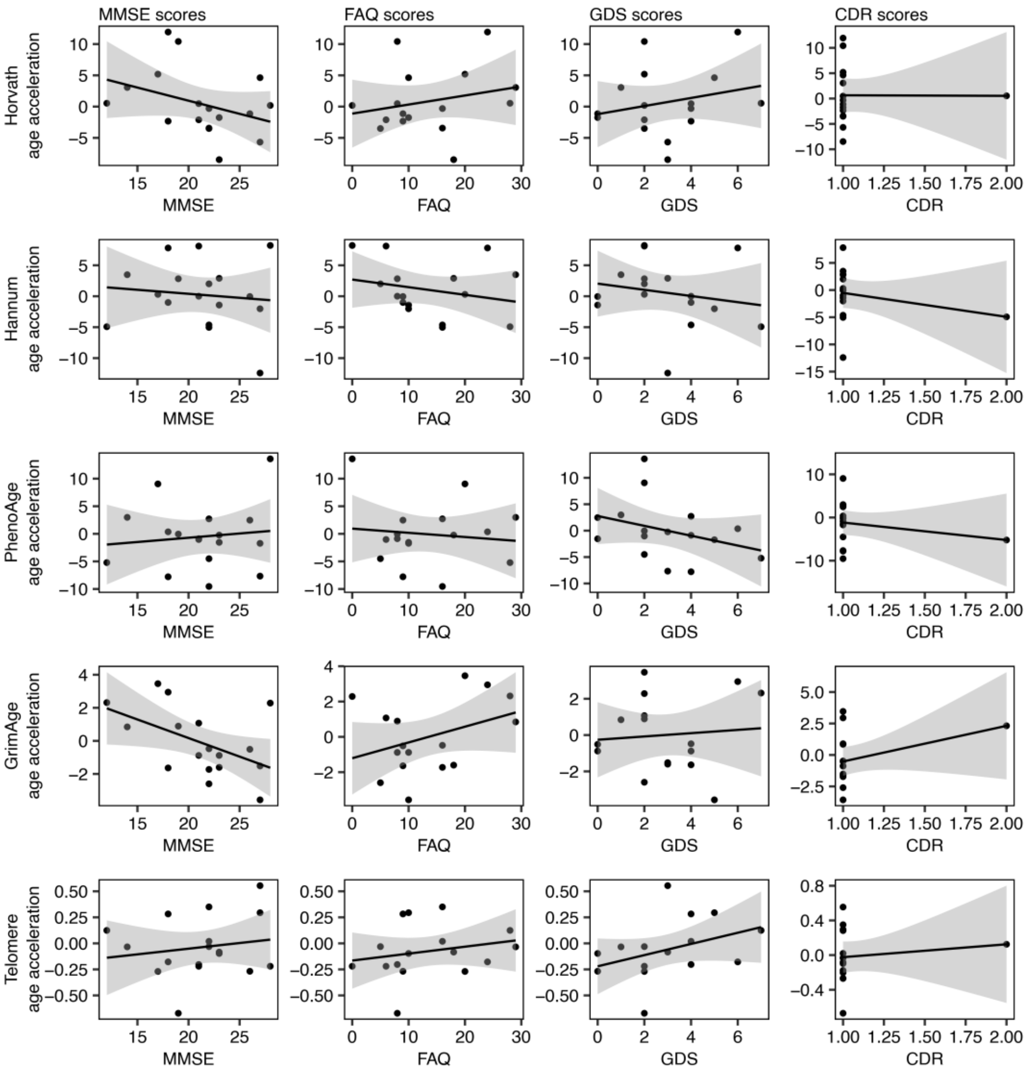

eFigure 9. Scatter plots showing the correlation between epigenetic age acceleration measurements for different epigenetic clocks (Horvath, Hannum, PhenoAge, GrimAge, Telomere) and neurological markers (MMSE, FAQ, GDS, CDR). across the study subjects belonging to the dementia timeL subgroup (i.e. those subjects diagnosed with dementia). The black lines indicate linear fit. No significant associations were observed after adjustment for multiple testing. MMSE: Mini Mental State Examination; FAQ: Functional Activities Questionnaire; GDS: Geriatric Depression Scale; CDR: Clinical Dementia Rating.
